# The efficacy and safety of *Serenoa repens* extract for the treatment of patients with chronic prostatitis/chronic pelvic pain syndrome: a multicenter, randomized, double-blind, placebo-controlled trial

**DOI:** 10.1007/s00345-020-03577-2

**Published:** 2021-01-16

**Authors:** Kai Zhang, Run-Qi Guo, Shan-Wen Chen, Bin Chen, Xin-Bo Xue, Shan Chen, Jian Huang, Ming Liu, Ye Tian, Li Zuo, Ming Chen, Li-Qun Zhou

**Affiliations:** 1 Department of Urology, Peking University First Hospital, The Institute of Urology, Peking University, National Urological Cancer Center, Beijing, 100034 China; 2 Beijing Key Laboratory of Urogenital Diseases (Male) Molecular Diagnosis and Treatment Center, Beijing, China; 3 Minimally Invasive Tumor Therapies Center, Beijing Hospital, National Center of Gerontology, Beijing, China; 4 Institute of Geriatric Medicine, Chinese Academy of Medical Sciences, Beijing, China; 5 Department of Urology, Huashan Hospital of Fudan University, Shanghai, China; 6 Department of Urology, Renji Hospital, Shanghai Jiaotong University School of Medicine, Shanghai, China; 7 Department of Urology, The Second Affiliated Hospital, Soochow University, Suzhou, China; 8 Department of Urology, Beijing Tongren Hospital, Capital Medical University, Beijing, China; 9 Department of Urology, Sun Yat-Sen Memorial Hospital, Sun Yat-Sen University, Guangzhou, Guangdong China; 10 Department of Urology, Beijing Hospital, Beijing, China; 11 Department of Urology, Beijing Friendship Hospital, Capital Medical University, Beijing, China; 12 Department of Urology, Changzhou No. 2 People’s Hospital Affiliated to Nanjing Medical University, Changzhou, Jiangsu China; 13 Department of Urology, Zhongda Hospital, Southeast University, Nanjing, 210000 Jiangsu China

**Keywords:** Chronic pelvic pain syndrome, Chronic prostatitis, Efficacy, Safety, *Serenoa repens*

## Abstract

**Purpose:**

To perform a placebo-controlled trial to evaluate the efficacy and safety of *Serenoa repens* extract (SRE) for the treatment of chronic prostatitis/chronic pelvic pain syndrome (CP/CPPS).

**Methods:**

We conducted a double-blind, randomized, placebo-controlled, multicenter, clinical phase 4 study of 221 patients with CP/CPPS across 11 centers. Participants were randomly assigned in a 2:1 ratio to receive SRE or placebo for 12 weeks. The primary efficacy endpoint was the change in total score on the National Institutes of Health-Chronic Prostatitis Symptom Index (NIH-CPSI). Secondary efficacy endpoints included improvements within each domain of NIH-CPSI, clinical response rate, and International Index of Erectile Function 5 items (IIEF-5).

**Results:**

In total, 226 patients were enrolled and randomized between January 2017 and June 2018. Of these 221 patients were included in the intent-to-treat analysis: 148 in the SRE group and 73 patients in the placebo group. Compared to the placebo, SRE led to statistically significant improvements in the NIH-CPSI total score and sub-scores. The significant improvements of NIH-CPSI scores were established after 2 weeks from the first dose, and continued to the end of the treatment. Furthermore, a significantly higher rate of patients achieved a clinical response in the SRE group compared with that in the placebo group (73.0% *vs* 32.9%, *P* < 0.0001). Only minor adverse events were observed across the entire study population.

**Conclusions:**

SRE was effective, safe, and clinically superior to placebo for the treatment of CP/CPPS. ChiCTR-IPR-16010196, December 21, 2016 retrospectively registered

**Supplementary Information:**

The online version contains supplementary material available at 10.1007/s00345-020-03577-2.

## Introduction

The prostatitis category III, as classified by the National Institutes of Health (NIH), and also named as chronic prostatitis/chronic pelvic pain syndrome (CP/CPPS), is a common disease with a complex and heterogeneous etiology [1, 2]. CP/CPPS is characterized by pain or discomfort in the pelvic region, often associated with urinary symptoms and/or sexual dysfunction, and psychologic consequences [3].

CPPS is not an anatomically or pathologically defined disease, and has multifactorial origin. The evidence-based treatments for CP/CPPS are not as adequate as other “prostate diseases” [4, 5]. Previous publications have reported the successful application of phytotherapeutic agents, such as pollen extract, quercetin, or saw palmetto/*Serenoa repens* (SR), with pain-alleviating effects [6–9]. Moderate-quality evidence indicates that phytotherapy probably causes a small decrease in prostatitis symptoms with few adverse events [10], however, the present literatures provide few powerful evidence for the recommendations of *Serenoa repens* extract (SRE) for CP/CPPS [5]. In addition, there has been rare reports on large-scale, placebo-controlled, SRE single-use trials. Therefore, we conducted a multicenter, randomized, double-blind, and placebo-controlled trial to evaluate the efficacy and safety of SRE in patients with CP/CPPS.

## Subjects and methods

### Study design and participants

This multicenter, randomized, double-blind, placebo-controlled study describes a clinical phase 4 trial conducted in 11 Chinese urologic centers between January 2017 and June 2018. The study protocol was approved by the Ethics Committee of Peking University First Hospital (Institutional Review Board Approval Number: 2016-038). This trial was reported based on the Consolidated Standards for Reporting Trials statement.

A total of 226 eligible patients were recruited from the outpatient departments of participating centers. The inclusion criteria were as follows: men, 18–50 years-of-age; pain or discomfort in the pelvic area for at least 3 months; total scores on the NIH Chronic Prostatitis Symptom Index (NIH-CPSI) > 10, and pain scores ≥ 4; clinically and laboratory diagnosed CP/CPPS; at least once sexual attempt in the previous 3 months. The exclusion criteria were as follows: urinary tract infection, acute epididymitis, or suspected prostate cancer; genital herpes; diseases that affected micturition; trauma or surgery that might affect the evaluation of drug efficacy; severe cardiovascular disease, sexually transmitted diseases, malignant tumors, peptic ulcers, or hemorrhage disease; current use of antibiotics, non-steroidal anti-inflammatory drugs, α-adrenergic blockers, bioflavonoids, Chinese patent medicines, plant drugs for prostatitis, or drugs that affected bladder function or sexual activity; liver or kidney insufficiency, or levels of aspartate transaminase or alanine transaminase that exceeded 1.5 times the upper limit of the normal range, or levels of creatinine that exceeded the upper limit of the normal range and were considered clinically significant; allergy to the study drug or similar drugs.

### Randomization and masking

Participants were randomly assigned in a 2:1 ratio to receive SRE or placebo treatment. Randomization and double blinding were performed in accordance with a randomization sequence created by a computer-generated program. Sequentially numbered study e-packs were securely stored at each study center using a password-protected computer database that was accessible by only the designer of the trial and a statistician. The investigator, participants, care providers, and those assessing clinical outcomes, were blinded to treatment assignment throughout the trial. The treatment and placebo capsules had the same appearance and taste.

### Procedures

Six visits were planned for each participant with CP/CPPS: V0, the screening visit (week − 2); V1, the first assessment, randomization and assignment (week 0, baseline); V2, the second assessment (week 2); V3, the third assessment (week 4); V4, the fourth assessment (week 8), and V5, the end-of-study assessment (week 12). At the start of the screening phase, we evaluated all patients by taking a detailed medical history, and assessing patients using both NIH-CPSI [11] and International Index of Erectile Function 5 (IIEF-5) [12] questionnaires. We also carried out a number of physical examinations, routine laboratory tests, ultrasound examination of the prostate, and a standardized four-glass test [13].

After a 2-week screening phase, participants were re-evaluated against the NIH-CPSI criteria. Those who satisfied the inclusion criteria were then randomly assigned to receive a daily treatment of either 320 mg of SRE (160 mg soft capsule BID; supercritical carbon dioxide extract, provided by TAD Pharma GmbH) or placebo (160 mg soft capsule BID). The double-blind treatment and follow-up phases lasted a total of 12 weeks. Antibiotics, non-steroidal anti-inflammatory drugs, α-adrenergic blockers, bioflavonoids, Chinese patent medicines, and other phytotherapeutic agents for CP/CPPS were forbidden throughout the study period.

### Outcomes

The primary efficacy endpoint was the change in NIH-CPSI total score (Q1–9) from baseline to that at each assessment visit. The secondary efficacy endpoints were improvements within the pain (Q1–4), urinary symptoms (Q5–6), and quality of life (QoL; Q7–9) domains of NIH-CPSI, and IIEF-5 score. As recommended by Nickel et al. [14], we introduced ‘clinical response’, defined as an improvement of the NIH-CPSI total score by at least 6 points. Safety was assessed primarily based on adverse event profiles.

### Statistical analysis

According to a phase 3 study of Cernilton [8], sample size was estimated on the basis of a mean reduction in NIH-CPSI score of 7.66 in the SRE group and 5.16 in the placebo group, with a standard deviation of 5.77, a power sensitivity (1 − *β*) of 80%, and a significance level of *α* = 0.025. Based on these calculations, we required at least 192 participants. Considering a drop-out rate of 10%, we enrolled 226 patients in this trial.

Descriptive data are reported as numbers (%) while continuous data are reported as mean ± standard deviation. We compared the entire cohort with respect to baseline characteristics, and then carried out exploratory subgroup analysis by categorizing patients into three sub-sets according to their baseline NIH-CPSI total scores: mild (11–14), moderate (15–29), and severe (30–43). Statistical analysis was performed using the Chi-squared test or by one-way analysis of variance (ANOVA) for categorical or continuous variables. Since there were only a small number of patients with NIH-CPSI of 11–14, we did not include these patients in our subgroup analyses. Changes of outcome over time were evaluated within and between groups with the Mann–Whitney *U* test or the Student’s *t* test as appropriate. A *P* < 0.05 was considered to be statistically significant. All statistical analysis were conducted with the SAS Computer Package Program version 9.1 (SAS Institute Inc., Cary, NC).

## Results

### Patient demographics

Of the 226 patients, 152 were randomly assigned to the SRE group and 74 to the placebo group. Two patients did not satisfy the criteria after re-evaluation and two patients withdrew consent for difficulty to comply with the study medication in the SRE group, whereas one patient was lost to follow-up from the first visit in the placebo group; thus, 221 patients were included in the intention-to-treat analysis (148 in the SRE group and 73 in the placebo group; SFigure1).

The demographic and baseline characteristics of those with CP/CPPS are shown in Table 1. There were no significant differences between the SRE group and the placebo group at the start of the treatment period with respect to NIH-CPSI (25.92 ± 8.17 vs 26.21 ± 7.85, respectively) and IIEF-5 scores (18.82 ± 4.47 vs 17.89 ± 5.50, respectively). STable1 shows the characteristics of patients with moderate or severe CP/CPPS.

**Table 1:** Demographic and baseline characteristic of study participants with CP/CPPS [intent-to-treat (ITT) analysis]

Variable	*Serenoa repens *(*n* = 148)	Placebo (*n* = 73)
Age (years, mean ± s.d.)	35.24 ± 7.85	32.84 ± 7.84
Weight (kg, mean ± s.d.)	69.77 ± 8.54	70.11 ± 10.30
Previous medication^a^, *n* (%)	43(29.05)	29(39.73)
Prostate irrelevant comorbidity, *n* (%)	8(5.41)	6(8.22)
Prostate size		
Length (cm, mean ± s.d.)	3.19 ± 0.76	3.09 ± 0.74
Width (cm, mean ± s.d.)	3.15 ± 0.50	3.09 ± 0.49
Height (cm, mean ± s.d.)	3.85 ± 0.69	3.99 ± 0.56
NIH-CPSI total score [Q1–9] (mean ± s.d.)	25.92 ± 8.17	26.21 ± 7.85
Pain domain [Q1–4]	13.21 ± 5.59	13.23 ± 5.35
Urinary symptoms domain [Q5–6] (mean ± s.d.)	4.58 ± 2.66	4.58 ± 2.71
QoL domain [Q7–9] (mean ± s.d.)	8.13 ± 2.18	8.40 ± 2.13
IIEF-5 (mean ± s.d.)	18.82 ± 4.47	17.89 ± 5.50

*CP/CPPS* chronic prostatitis/chronic pelvic pain syndrome, *NIH-CPSI* National Institute of Health Chronic Prostatitis Symptom Index, *PSA* prostate specific antigen, *QoL* quality of life, *IIEF-5* International Index of Erectile Function 5 items, *s.d.* standard deviation

^a^Antibiotics, non-steroidal anti-inflammatory drugs, α-adrenergic blockers, bioflavonoids, Chinese patent medicines and plant drugs for prostatitis

### Changes from baseline in the NIH-CPSI

Both the SRE group and the placebo group showed obvious improvements in NIH-CPSI (SFigure2A). After 2 weeks, patients with moderate CP/CPPS showed significant improvement with respect to the NIH-CPSI when compared to the placebo group (2.93 ± 3.12 vs 1.51 ± 3.70, *P* = 0.0257; STable2, SFigure3A). A similar improvement was observed in patients with severe CP/CPPS after 4 weeks of treatment (7.80 ± 6.88 vs 4.41 ± 4.33, *P* = 0.0250; STable2, SFigure4A). The proportion (%) of patients who demonstrated a six-point reduction in NIH-CPSI total score was a significantly higher in the SRE group than in the placebo group (73.0% vs 32.9%, *P* < 0.0001; Table 2).

**Table 2:** Efficacy outcomes after treatment for participants with CP/CPPS [intent-to-treat (ITT) analysis]

	*Serenoa repens *(*n* = 148) Mean ± s.d. or *n* (%)	Placebo (*n* = 73) Mean ± s.d. or n (%)	Mean difference or risk difference (95% CI)	*P* value
NIH-CPSI total score [Q1–Q9]				
V1–V2	3.46 ± 5.10	2.07 ± 4.03	1.39 (0.15, 2.63)	0.0433
V1–V3	5.30 ± 5.16	3.01 ± 4.14	2.29 (1.03, 3.55)	0.0011
V1–V4	7.49 ± 5.62	4.10 ± 5.55	3.39 (1.83, 4.95)	< 0.0001
V1–V5	9.39 ± 6.84	5.21 ± 6.1	4.18 (2.40, 5.96)	< 0.0001
Score of pain domain [Q1–Q4]				
V1–V2	1.96 ± 3.56	1.40 ± 3.04	0.56 (− 0.34, 1.46)	0.2488
V1–V3	3.02 ± 3.74	1.99 ± 3.03	1.03 (0.11, 1.95)	0.0413
V1–V4	4.11 ± 4.06	2.78 ± 3.88	1.33 (0.23, 2.43)	0.0213
V1–V5	5.07 ± 4.64	3.25 ± 4.13	1.82 (0.61, 3.03)	0.0049
Score of urinary symptoms domain [Q5–6]				
V1–V2	0.80 ± 1.52	0.19 ± 1.50	0.61 (0.19, 1.03)	0.0044
V1–V3	1.14 ± 1.56	0.38 ± 1.50	0.76 (0.33, 1.19)	0.0008
V1–V4	1.56 ± 1.85	0.45 ± 1.59	1.11 (0.64, 1.58)	< 0.0001
V1–V5	1.93 ± 2.02	0.60 ± 1.65	1.33 (0.83, 1.83)	< 0.0001
Score of QoL domain [Q7–9]				
V1–V2	0.70 ± 1.43	0.48 ± 1.38	0.22 (− 0.17, 0.61)	0.2693
V1–V3	1.15 ± 1.57	0.64 ± 1.56	0.51 (0.07, 0.95)	0.0250
V1–V4	1.82 ± 1.71	0.86 ± 1.69	0.96 (0.48, 1.44)	0.0001
V1–V5	2.40 ± 2.08	1.36 ± 2.97	1.04 (0.28, 1.80)	0.0005
Clinical response				
6-point decrease in NIH-CPSI	108 (73.0%)	24 (32.9%)	0.40 (0.27, 0.53)	< 0.0001
IIEF-5				
V5–V0	1.32 ± 2.95	1.01 ± 3.07	0.31 (− 0.54, 1.16)	0.4778

*CP/CPPS* chronic prostatitis/chronic pelvic pain syndrome, *NIH-CPSI* National Institute of Health Chronic Prostatitis Symptom Index, *QoL* quality of life, IIEF-5 International Index of Erectile Function 5 items, *CI* confidence interval, *s.d.* standard deviation, *V0* screening phase, *V1* visit at baseline, *V2* visit after 2 weeks, *V3* visit after 4 weeks, *V4* visit after 8 weeks, *V5* visit after 12 weeks

The pain domain decreased significantly from 13.21 to 8.14 in the SRE group and from 13.23 to 9.99 in the placebo group (SFigure2B). Similar decrease was also observed in patients with moderate or severe CP/CPPS (SFigure3B, 4B). The mean change from baseline was significantly higher in the SRE group compare to placebo after 4 weeks of treatment (3.02 ± 3.74 vs 1.99 ± 3.03, *P* = 0.0413; Table 2).

The urinary symptoms domain improved in both groups (SFigure2C, 3C, 4C). A significantly higher improvement in the SRE group compared to placebo could be first observed after 2 weeks (0.80 ± 1.52 vs 0.19 ± 1.50, *P* = 0.0044; Table 2) and across the entire treatment period.

The QoL domain also improved in both groups (SFigure2D, 3D, 4D). A tendency in favor of SRE was statistically significant after 4 weeks (1.15 ± 1.57 vs 0.64 ± 1.56, *P* = 0.0250; Table 2).

### Changes from baseline in the IIEF-5

Erectile function improved in both groups after 12 weeks (SFigure2E), and no significant difference was observed between the two groups (1.32 ± 2.95 vs 1.01 ± 3.07, *P* = 0.4778; Table 2). Nevertheless, when confined to moderate or severe CP/CPPS, it only improved significantly in the SRE group (SFigure3E, 4E). In addition, patients with severe CP/CPPS who received SRE for 12 weeks showed a significant improvement on IIEF-5 (2.31 ± 2.95 vs 0.89 ± 2.65, *P* = 0.0437; STable2).

### Safety

There were no significant differences with regards to the number of patients experiencing adverse events during the treatment period when compared between the two groups [four out of 152 patients in the treatment group (2.63%) vs three out of 74 patients in the placebo group (4.05%); *P* = 0.686]. There were no deaths recorded during the treatment period, and no adverse events that were considered to be serious.

Treatment-emergent adverse events were shown as follows: in the placebo group, two patients with nausea and stomach discomfort, and one with pruritus; in the *Serenoa repens* treatment group, two patients with nausea and stomach discomfort, one with hypertension, and one with lumbago.

## Discussion

Phytotherapy such as pollen extract is an organ specific treatment in UPOINT system [15], and was widely used in China [16]. SR, is another promising option for the management of CP/CPPS, largely because of a low incidence of side-effects and affirmative effects in patients suffering from lower urinary tract symptoms/benign prostatic hyperplasia (LUTS/BPH) [17]. However, very few of phytotherapeutic agents have been verified in prospective controlled clinical trials in patients with CP/CPPS. In the present study, SRE had a significant effect on pain relief, while also improving urinary symptoms and QoL.

A previous 1-year treatment comparing saw palmetto and finasteride in 64 consecutive men with CPPS suggested that saw palmetto improving the CPPS symptoms at 3 months and then the efficacy diminished [18]. It is important to note that extracts of SR from different suppliers may not exhibit the same biological or clinical effects [17]. According to a network meta-analysis, a hexanic lipidosterolic extract prepared from SR was associated with better improvement than non-hexanic extracts, at least in regards to the alleviation of LUTS [19]. Consequently, it is not possible to extrapolate the effects of one brand to another.

In this study, compared with the placebo group, the SRE group showed significant improvements in both the NIH-CPSI total score and the urinary symptoms domain score when assessed just 2 weeks after the first dose; furthermore, the improvements above continued over time. The urinary symptoms domain appeared to make the most significant contribution to the NIH-CPSI; similar observations were reported in another study evaluating the effects of SRE in patients with LUTS/BPH [17]. According to early findings reported by the Medical Therapies of Prostate Symptoms (MTOPS) study and the REDUCE (Reduction by Dutasteride of Prostate Cancer Events) population, there are evidences of chronic prostate inflammation being relevant with LUTS [20, 21], and chronic inflammation was also associated with symptom progression in patients with CP/CPPS [22]. In a randomized biopsy study, SR treatment was reported to reduce prostatic inflammation, as determined by the measurement of T-lymphocyte markers, B-lymphocyte markers, and macrophage markers [23]. Furthermore, it has also been reported that SR exhibits α1-adrenoceptor-inhibitory properties [24]; in combination with its chronic anti-inflammatory ability, these properties may facilitate the continuous improvement in urinary symptoms. A recent systematic review and meta-analysis found that SR could effectively increase urinary flow and improve urinary symptoms, and exhibited comparable efficacy to tamsulosin and short-term 5 α-reductase inhibitors in terms of relieving LUTS [25].

The pelvic pain is the main symptom and primary feature of CP/CPPS, which may be often triggered in both urination and ejaculation episodes, with negatively impact on LUTS, sexual activity, and then QoL [26]; consequently, pain relief is a predominate feature for the management of patients with CP/CPPS. Nevertheless, no single etiological explanation has been put forward to account for the pelvic pain. According to a recent comparative study, carried out both in vitro and in vivo, SR exhibited similar levels of efficacy in reducing nuclear factor-kappa B binding activity, and inhibiting the expression of cyclooxygenase and prostaglandin, both of which are known to be involved in inflammatory pain [27].

Besides, SRE was significantly improved erectile function at the end of the treatment. In a study involving both rat and rabbit models, SR was shown to exhibit significant potential for the prevention or treatment of erectile dysfunction by increasing the expression levels of inducible nitric oxide synthase and inhibiting phosphodiesterase 5 activity in corpus cavernosum smooth muscles [28].

We observed an obvious placebo effect, as the NIH-CPSI total score decreased significantly after 12 weeks in both the treatment and placebo groups (Supplementary Fig. 2A). This implies CP/CPPS might be a self-healing condition in a portion of patients. We hypothesize that this might be hint with psychosocial effects, which may lead to improvements in the QoL. However, the differences between the two groups became significant after 2–4 weeks, suggesting that a longer treatment period is required for patients with severe CP/CPPS. It is interesting that the clinical response, as defined by a six-point improvement of the NIH-CPSI total score at the end of the trial, was seen in 73.0% of the patients receiving SRE, but in only 32.9% of patients in the placebo group (Table 2). This observation provides robust confirmation of the efficacy of SRE.

To the best of our knowledge, this is the largest multicenter, randomized, double-blind, placebo-controlled study to investigate the efficacy and safety of phytotherapy in patients with CP/CPPS. We confirm that SRE is effective and well-tolerated in patients with CP/CPPS. At present, there is still controversy with regards to the treatment of such patients with α-adrenergic blockers [29], largely because such preparations are associated with a number of side effects, including orthostatic hypotension, erectile and/or ejaculate dysfunction [30]. Consequently, it is possible that SRE might provide a better option or substitution for such patients, with a more favorable benefit/risk consideration.

However, this study has some limitations. First, we used a standard dose of SRE (320 mg); this is also the dose used to treat LUTS/BPH. Second, we lacked adequate data for mild CP/CPPS patients. Moreover, we did not distinguish between patients with or without inflammatory CP/CPPS. Finally, we did not follow-up the patients after the end of treatment to determine if the effects continued without SRE, although our follow-up time period was in line with other similar studies [8].

## Conclusions

The SRE was effective, and clinically superior to placebo for CP/CPPS, improving pain as well as urinary symptoms, QoL with few side effects.

## Supplementary Information

Below is the link to the electronic supplementary material.

Supplementary file1 (TIFF 2317 KB)

Supplementary file2 (PDF 2686 KB)

Supplementary file3 (PDF 2780 KB)

Supplementary file4 (PDF 2740 KB)

Supplementary file5 (DOCX 17 KB)

Supplementary file6 (DOCX 18 KB)

Supplementary file7 (DOC 218 KB)

## Data Availability

All original data are available in Peking University First Hospital.
