# Supplementary figures and images for: The efficacy and safety of Serenoa repens extract for the treatment of patients with chronic prostatitis/chronic pelvic pain syndrome: a multicenter, randomized, double-blind, placebo-controlled trial
Source: World J Urol. 2021 Jan 16;39(9):3489–95. doi: 10.1007/s00345-020-03577-2 (PMC8510895; doi:10.1007/s00345-020-03577-2)

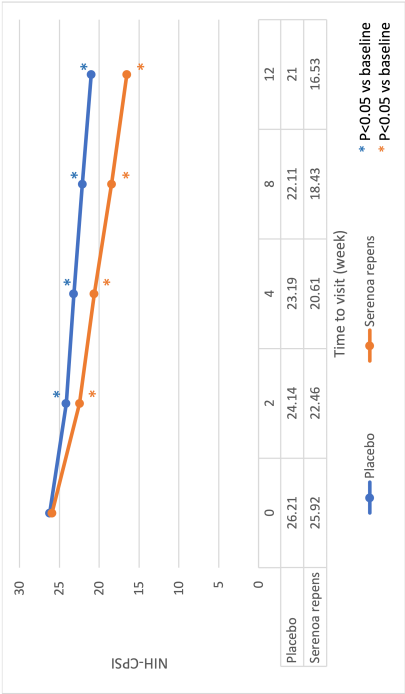

A

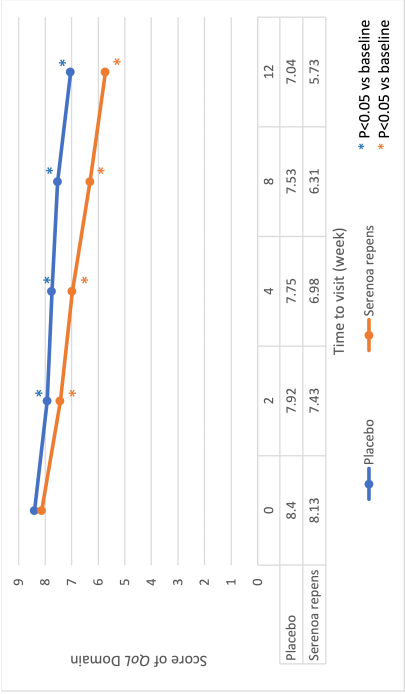

D

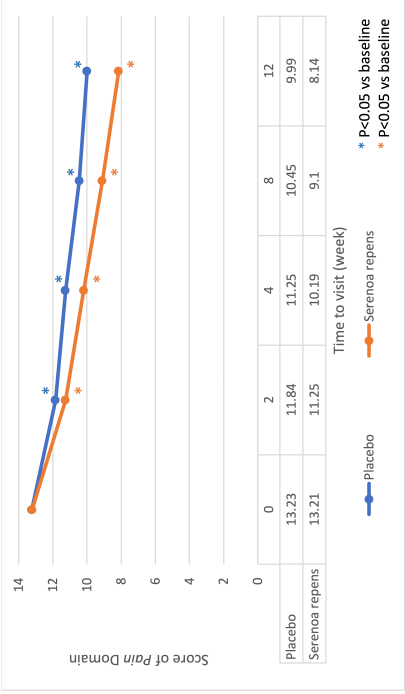

B

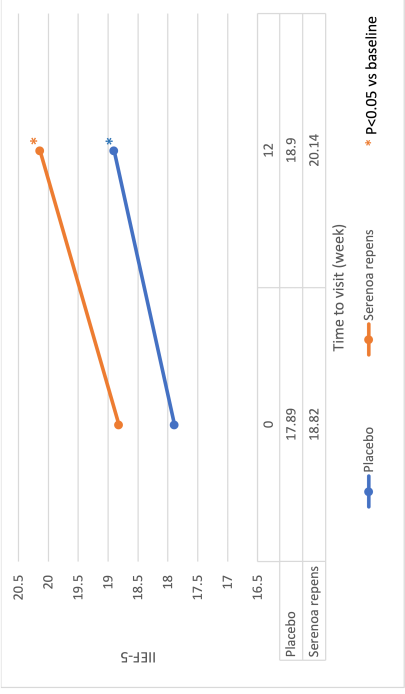

E

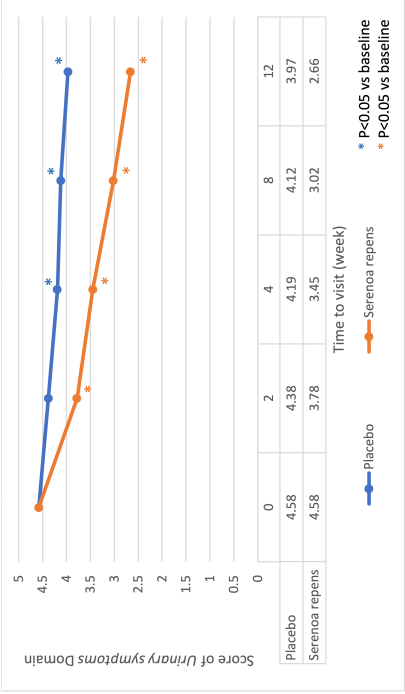

C

Supplement: Supplementary file 2 — Supplementary file2 (PDF 2686 KB) [file 345_2020_3577_MOESM2_ESM.pdf]

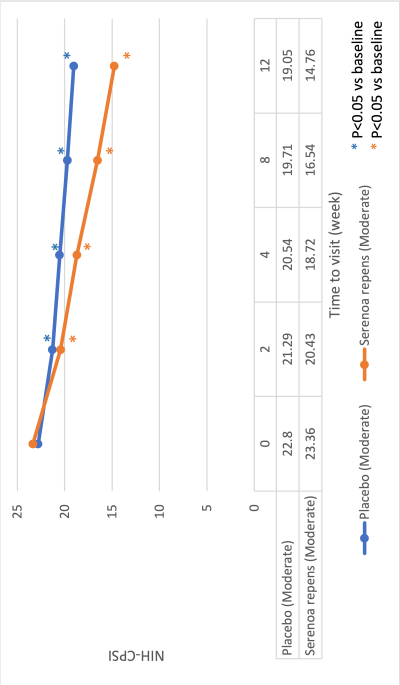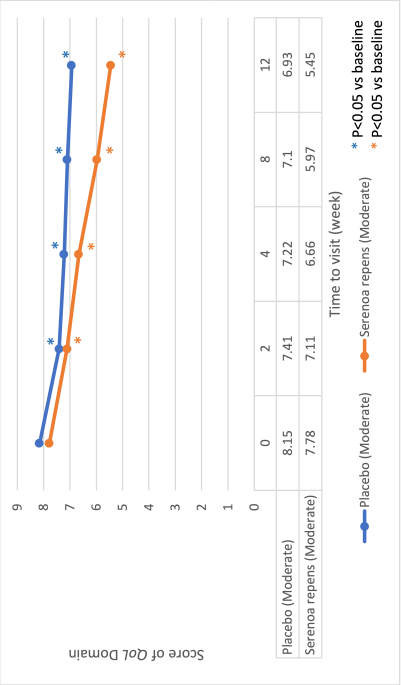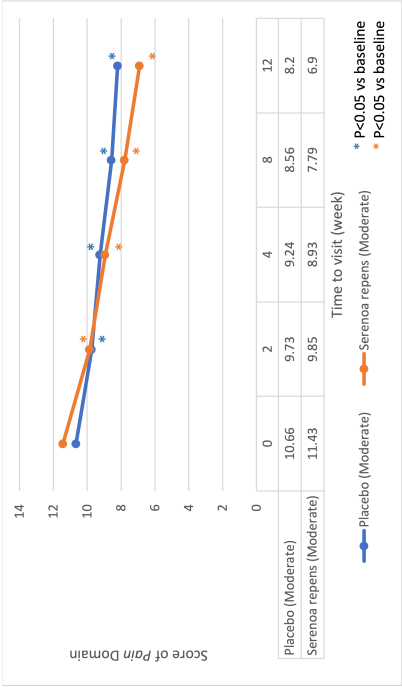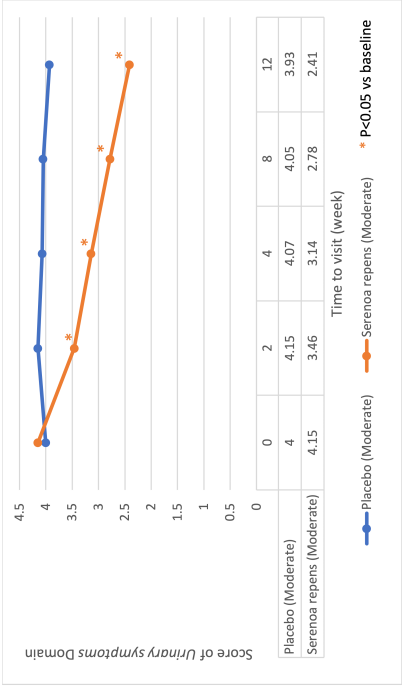

A

B

C

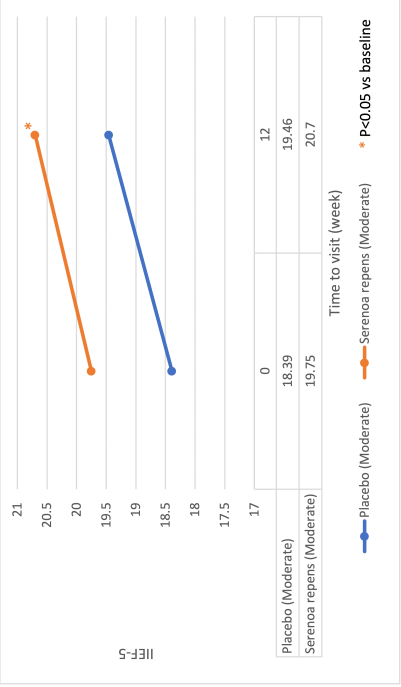

D

E

Supplement: Supplementary file 3 — Supplementary file3 (PDF 2780 KB) [file 345_2020_3577_MOESM3_ESM.pdf]

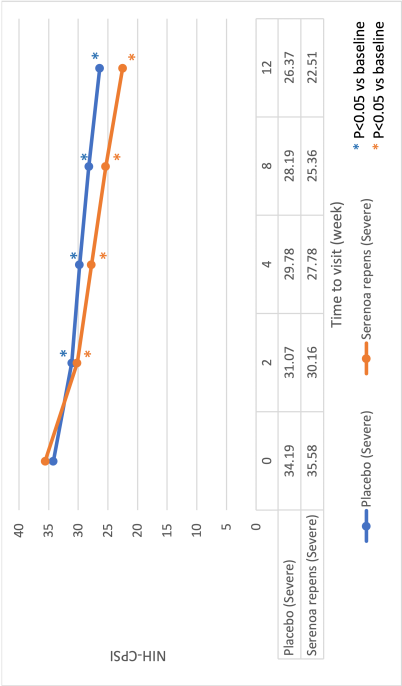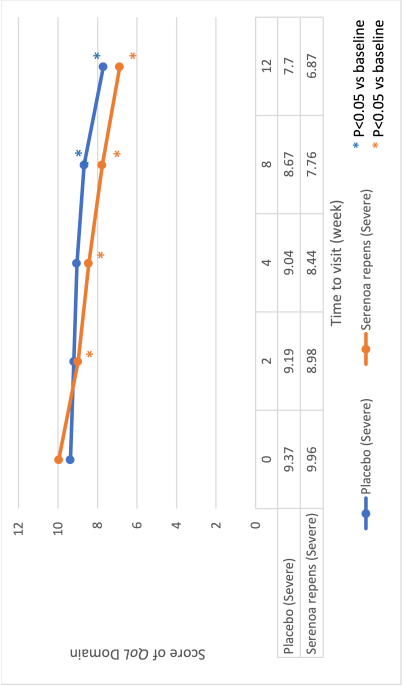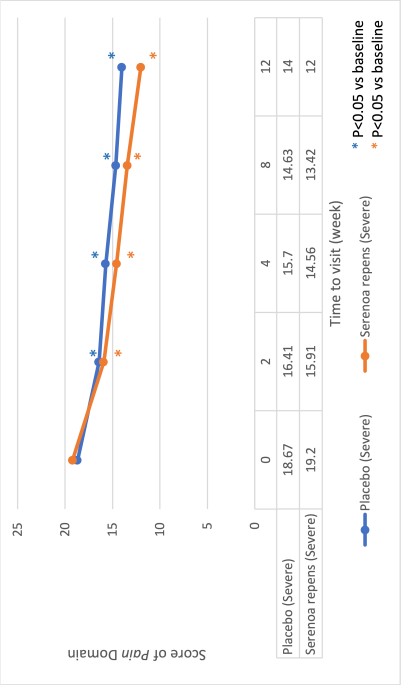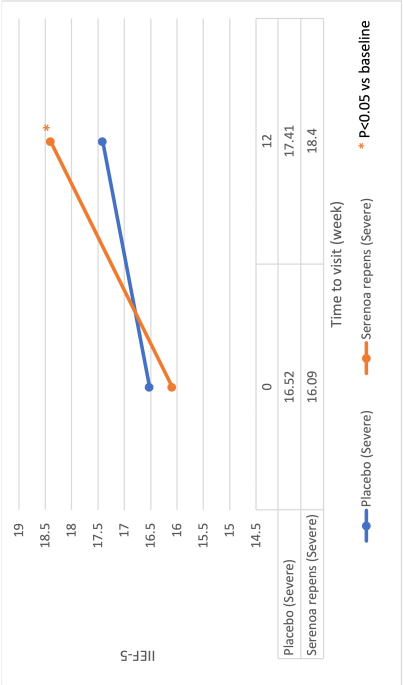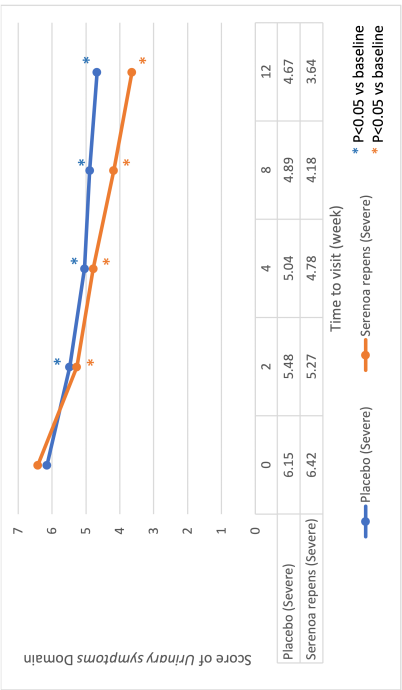

Supplement: Supplementary file 4 — Supplementary file4 (PDF 2740 KB) [file 345_2020_3577_MOESM4_ESM.pdf]
