# Supplementary material for: The efficacy and safety of Serenoa repens extract for the treatment of patients with chronic prostatitis/chronic pelvic pain syndrome: a multicenter, randomized, double-blind, placebo-controlled trial
Source: World J Urol. 2021 Jan 16;39(9):3489–95. doi: 10.1007/s00345-020-03577-2 (PMC8510895; doi:10.1007/s00345-020-03577-2)
Supplement: Supplementary file 5 — Supplementary file5 (DOCX 17 KB) [file 345_2020_3577_MOESM5_ESM.docx]

| **STable1 - Demographic and baseline characteristic of study participants with moderate or severe CP/CPPS (intent-to-treat [ITT] analysis)** | | | | | |
| --- | --- | --- | --- | --- | --- |
| Variable | NIH-CPSI 15-29 | |  | NIH-CPSI 30-43 | |
|  | *Serenoa repens*  (n=87) | Placebo  (n=41) |  | *Serenoa repenss*  (n=45) | Placebo  (n=27) |
| Age (years, mean±s.d.) | 35.46±8.12 | 32.61±8.16 |  | 36.24±7.66 | 34.07±7.71 |
| Weight (kg, mean±s.d.) | 69.77±7.56 | 70.66±11.26 |  | 69.56±10.10 | 69.65±9.74 |
| Previous medication^✝^, n(%) | 27(31.03) | 17(41.46) |  | 13(28.89) | 10(37.04) |
| Prostate irrelevant comorbidity, n(%) | 5(5.75) | 1(2.4) |  | 3(6.67) | 5(18.52) |
| Prostate size |  |  |  |  |  |
| length (cm, mean±s.d.) | 3.12±0.72 | 3.17±0.71 |  | 3.14±0.79 | 2.88±0.71 |
| width (cm, mean±s.d.) | 3.14±0.44 | 3.02±0.46 |  | 3.12±0.61 | 3.13±0.53 |
| height (cm, mean±s.d.) | 3.98±0.73 | 3.96±0.56 |  | 3.70±0.61 | 4.03±0.61 |
| NIH-CPSI total score [Q1-9] (mean±s.d.) | 23.36±3.70 | 22.80±3.97 |  | 35.58±4.85 | 34.19±4.20 |
| *Pain* domain [Q1-4] | 11.43±2.94 | 10.66±3.02 |  | 19.20±18.67 | 18.67±3.33 |
| *Urinary symptoms* domain [Q5-6] (mean±s.d.) | 4.15±2.22 | 4.00±2.33 |  | 6.42±2.55 | 6.15±2.46 |
| *QoL* domain [Q7-9] (mean±s.d.) | 7.78±1.71 | 8.15±2.07 |  | 9.96±1.24 | 9.37±1.71 |
| IIEF-5 (mean±s.d.) | 19.75±3.59 | 18.39±4.22 |  | 16.09±5.36 | 16.52±7.18 |
| CP/CPPS = chronic prostatitis/chronic pelvic pain syndrome; NIH-CPSI = National Institute of Health Chronic Prostatitis Symptom Index; QoL = quality of life; IIEF-5 = International Index of Erectile Function 5 items; s.d. = standard deviation.  ^✝^ Antibiotics, non-steroidal anti-inflammatory drugs, α-adrenergic blockers, bioflavonoids, Chinese patent medicines and plant drugs for prostatitis. | | | | | |
