# Supplementary material for: The efficacy and safety of Serenoa repens extract for the treatment of patients with chronic prostatitis/chronic pelvic pain syndrome: a multicenter, randomized, double-blind, placebo-controlled trial
Source: World J Urol. 2021 Jan 16;39(9):3489–95. doi: 10.1007/s00345-020-03577-2 (PMC8510895; doi:10.1007/s00345-020-03577-2)
Supplement: Supplementary file 6 — Supplementary file6 (DOCX 18 KB) [file 345_2020_3577_MOESM6_ESM.docx]

| **STable2 – Efficacy outcomes after treatment for participants with moderate or severe CP/CPPS (Intent-to-treat [ITT] analysis)** | | | | | | | | | |
| --- | --- | --- | --- | --- | --- | --- | --- | --- | --- |
|  | NIH-CPSI 15-29 | | | |  | NIH-CPSI 30-43 | | | |
|  | *Serenoa repens*  (n=87)  mean±s.d.  or n (%) | Placebo  (n=41)  mean±s.d. or n (%) | Mean difference  or  risk difference  (95% CI) | *P* value |  | *Serenoa repens*  (n=45)  mean±s.d.  or n (%) | Placebo  (n=27)  mean±s.d. or n (%) | Mean difference  or  risk difference  (95% CI) | *P* value |
| NIH-CPSI total score [Q1-Q9] |  |  |  |  |  |  |  |  |  |
| V1-V2 | 2.93±3.12 | 1.51±3.70 | 1.42 (0.11, 2.73) | 0.0257 |  | 5.42±7.64 | 3.11±4.69 | 2.31 (-0.54, 5.16) | 0.1606 |
| V1-V3 | 4.63±3.81 | 2.27±3.99 | 2.36 (0.9-3.82) | 0.0016 |  | 7.80±6.88 | 4.41±4.43 | 3.47 (0.86, 6.08) | 0.0250 |
| V1-V4 | 6.82±4.20 | 3.10±5.52 | 3.72 (1.81, 5.63) | <0.0001 |  | 10.22±7.33 | 6.00±5.66 | 4.22 (1.20, 7.24) | 0.0124 |
| V1-V5 | 8.60±4.92 | 3.76±5.02 | 4.84 (2.99, 6.69) | <0.0001 |  | 13.07±8.76 | 7.81±7.34 | 5.26 (1.93, 8.59) | 0.0110 |
| Score of *pain* domain [Q1-Q4] |  |  |  |  |  |  |  |  |  |
| V1-V2 | 1.57±2.11 | 0.93±2.24 | 0.64 (-0.18, 1.46) | 0.1140 |  | 3.29±5.35 | 2.26±4.08 | 1.03 (-1.16, 3.22) | 0.3926 |
| V1-V3 | 2.49±2.64 | 1.41±2.57 | 1.08 (0.12, 2.04) | 0.0314 |  | 4.64±5.23 | 2.96±3.67 | 1.68 (-0.38, 3.74) | 0.1473 |
| V1-V4 | 3.63±2.81 | 2.10±3.77 | 1.53 (0.23, 2.83) | 0.0113 |  | 5.78±5.73 | 4.04±4.15 | 1.74 (-0.55, 4.03) | 0.1735 |
| V1-V5 | 4.53±3.21 | 2.46±3.19 | 2.07 (0.88, 3.26) | 0.0009 |  | 7.20±6.39 | 4.67±5.29 | 2.53 (-0.20, 5.26) | 0.0877 |
| Score of *urinary symptoms* domain [Q5-6] |  |  |  |  |  |  |  |  |  |
| V1-V2 | 0.69±1.40 | -0.15±1.30 | 0.84 (0.35, 1.33) | 0.0016 |  | 1.16±1.86 | 0.67±1.44 | 0.49 (-0.28, 1.26) | 0.2456 |
| V1-V3 | 1.01±1.39 | -0.07±1.33 | 1.08 (0.58, 1.58) | 0.0001 |  | 1.64±1.84 | 1.11±1.60 | 0.53 (-0.28, 1.34) | 0.2154 |
| V1-V4 | 1.37±1.81 | 0.05±1.32 | 1.32 (0.77, 1.87) | <0.0001 |  | 2.24±1.96 | 1.26±1.79 | 0.98 (0.09, 1.87) | 0.0363 |
| V1-V5 | 1.74±1.87 | 0.07±1.35 | 1.67 (1.10, 2.24) | <0.0001 |  | 2.78±2.22 | 1.48±1.85 | 1.30 (0.35, 2.25) | 0.0131 |
| Score of *QoL* domain [Q7-9] |  |  |  |  |  |  |  |  |  |
| V1-V2 | 0.67±1.18 | 0.73±1.67 | -0.06 (-0.63, 0.51) | 0.8004 |  | 0.98±1.78 | 0.19±0.83 | 0.79 (0.18, 1.40) | 0.0331 |
| V1-V3 | 1.13±1.41 | 0.93±1.82 | 0.2 (-0.43, 0.83) | 0.4991 |  | 1.51±1.78 | 0.33±1.14 | 1.18 (0.51, 1.85) | 0.0030 |
| V1-V4 | 1.82±1.69 | 1.05±1.91 | 0.77 (0.09, 1.45) | 0.0235 |  | 2.20±1.73 | 0.70±1.44 | 1.50 (0.76, 2.24) | 0.0003 |
| V1-V5 | 2.33±1.88 | 1.22±2.09 | 1.11 (0.36, 1.86) | 0.0031 |  | 3.09±2.32 | 1.67±1.94 | 1.42 (0.42, 2.42) | 0.0095 |
| Clinical response |  |  |  |  |  |  |  |  |  |
| 6-point decrease in NIH-CPSI | 65(74.7%) | 11(26.8%) | 0.48 (0.32, 0.64) | <0.0001 |  | 37(82.2%) | 13(48.1%) | 0.34 (0.12, 0.56) | 0.0020 |
| IIEF-5 |  |  |  |  |  |  |  |  |  |
| V5-V0 | 0.95±3.05 | 1.07±3.50 | -0.12 (-1.37, 1.13) | 0.8444 |  | 2.31±2.95 | 0.89±2.65 | 1.42 (0.10, 2.74) | 0.0437 |
| CP/CPPS = chronic prostatitis/chronic pelvic pain syndrome; NIH-CPSI = National Institute of Health Chronic Prostatitis Symptom Index; QoL = quality of life; IIEF-5 = International Index of Erectile Function 5 items; CI = confidence interval; s.d. = standard deviation; V0 = screening phase; V1 = visit at baseline; V2 = visit after 2 weeks; V3 = visit after 4 weeks; V4 = visit after 8 weeks; V5 = visit after 12 weeks. | | | | | | | | | |
